# Response to: Low gestational age is associated with less anastomotic complications after open primary repair of esophageal atresia with tracheoesophgeal fistula. *BMC Paediatric* 2020; 20:267

**DOI:** 10.1186/s12887-021-02900-z

**Published:** 2021-09-25

**Authors:** V. Coles, I. Yardley

**Affiliations:** 1 Department of Paediatric Surgery, Evelina London Children’s Hospital, London, UK; 2 Faculty of Life Sciences and Medicine, King’s College London, London, UK

Dear Editor,

We read with interest Dingermann et al.’s recent paper *Low gestational age is associated with less anastomotic complications after open primary repair of esophageal atresia with tracheoesophageal fistula*. The authors and their units should be commended on achieving zero operative mortality and a remarkably low complication rate in a cohort including some very small babies. The finding of a decreased risk of complications in patients born at a lower gestational age, a group traditionally felt to be at higher risk, is a surprising one with no obvious biologically plausible explanation and therefore warrants further scrutiny [1, 2]. We have some concerns over the way the data have been processed and analysed and consequently feel the conclusions drawn should be reconsidered.

The first decision made that is questionable is the decision to set the cut off at 34 weeks gestational age for the two groups. This is surprising as the usually accepted definition of prematurity is a baby born at under 37 completed weeks of gestation. References are given to support the choice of a 34-week cut off but one of these is from an ethics textbook [3] and another, concerning the administration of maternal steroids to promote fetal lung maturation, itself uses 37 weeks as the definition of prematurity [4]. As the median gestational age in the cohort presented was 37 weeks, using this widely accepted definition of “preterm” would have given two equal groups to compare. Insufficient data is given in the paper to make a definitive assessment but it appears from Fig. 2 that using 37 weeks would give roughly equal allocations of complications to the term and preterm groups.

The key analysis comparing the occurrence of complications (anastomotic leak, recurrent fistula and anastomotic stricture) in each group (< 34 weeks and ≥ 34 weeks gestation) has been performed in a highly unusual manner. These are clearly categorical data (yes/no for the occurrence of each complication) and we would expect the analysis to be with the Chi-squared or Fisher’s Exact Test if numbers were small. Instead, an unpaired t-test has been used, apparently on the proportions of complications in each group. How a range of proportions with the mean and standard deviation needed to carry out a t-test have been generated for each group is unclear. When the analysis is performed using more standard statistical methods, the findings are rather different. Using a Fisher’s Exact Test and a threshold of *P* < 0.05 to re-run the analysis for Table 3, there was no statistically significant difference seen between the two groups for any of the reported surgical complications (Table 3: amended). Unsurprisingly, there were statistically significant differences in the incidence of respiratory distress syndrome and intraventricular haemorrhage between those under 34 weeks gestation and those over, [4, 5], supporting the use of Fisher’s Exact Test.

**Table 3:** (amended): Postoperative outcome after primary esophageal anastomosis for eophageal atresia with distal tracheoesophageal fistula

**Complications**		**Patients < 34 GA (*n* = 20)**	**Patients ≥ 34 GA (*n* = 55)**	***P* value (Fisher’s Exact Test)**
**Related to surgical intervention**	Anastomotic leakage	0	3	0.5602
	Recurrent fistula	0	3	0.5602
	Anastomotic stricture	0	8	0.1000
**Related to prematurity**	Infant respiratory distress syndrome	11	0	< 0.0001
	Intraventricular bleeding	5	2	0.0127

A subsequent analysis (Fig. 2) again uses percentages of patients experiencing complications at each completed week of gestational age and a correlation coefficient has been calculated. This method seems unsound for two reasons. The first is that the proportions (percentages) used are the sum of all complications. Given that the occurrence of an anastomotic leak increases the risk of both a recurrent fistula and anastomotic stricture, this is likely to represent double or even triple counting of the same complications. Secondly, these are again categorical events and so trying to construct a correlation line is an inappropriate statistical method.

Lastly, the authors note a significant difference between the two groups in the presence of associated anomalies in addition to the esophageal atresia and trachea-esophageal fistula. These associated anomalies are known to be linked with an increased incidence of complications [6, 7]. Despite this, no multi-variate analysis has been attempted, as a minimum a two-factor regression model would be needed although we acknowledge the limitations of these models with small numbers.

On reading the reviewers comments, which *BMC Pediatrics* commendably make available on line, neither peer reviewer comments on the statistical methods employed although both state in their review submissions “I am able to assess the statistics”. It would seem that their review and assessment was insufficiently robust to ensure the veracity of what has been published. The data presented in the paper do not support the conclusion that low gestational age is associated with less anastomotic complications after open primary repair of esophageal atresia.

## Data Availability

All data generated or analysed during this study are included in this published article.
